# Successful treatment with alectinib after crizotinib-induced hepatitis in ALK-rearranged advanced lung cancer patient: a case report

**DOI:** 10.1186/s12890-020-01390-6

**Published:** 2021-01-28

**Authors:** Flávia Amaral Duarte, Leonardo Brand Rodrigues, Flávia Rocha Paes, Paulo Henrique Costa Diniz, Helena Flávia Cuba de Almada Lima

**Affiliations:** 1 Department of Clinical Oncology, Oncoclinicas Group, Oncocentro, Rua Roma 561, 4th floor, Santa Lúcia, Belo Horizonte, MG 30360-680 Brazil; 2 Department of Thoracic Surgery, Madre Teresa Hospital, Av. Raja Gabáglia, 1002, Gutierrez, Belo Horizonte, MG 30380-090 Brazil

**Keywords:** Crizotinib, Hepatotoxicity, Alectinib, Non-small cell lung cancer, Case report

## Abstract

**Background:**

Besides the clinical benefit of crizotinib in ALK-rearranged metastatic non-small cell lung cancer (NSCLC), concerns about its hepatotoxicity have arisen. It is not clear whether this is a drug class side effect or if the use of other selective ALKs inhibitors is safe after this serious adverse event. While evidence from clinical trials is scarce, reports of treatment after crizotinib-induces hepatitis may add to clinical decision.

**Case presentation:**

Herein, we report a case of acute hepatitis induced by crizotinib in a 32-years-old female diagnosed with metastatic NSCLC, harboring the ALK-rearrangement. After 60 days of crizotinib therapy, the patient presented with acute hepatitis, diagnosed after investigation of non-specific symptoms, such as nausea and fatigue. Serum aspartate aminotransferase and alanine aminotransferase levels had increased from baseline to 3010 IU/L and 9145 IU/L, respectively. Total bilirubin increased up to 7.91 mg/dL, but she did not develop liver failure. After crizotinib discontinuation, a gradual hepatic function recovery occurred. Unfortunately, during the period without specific oncology treatment, her disease showed an unequivocal progression. Therefore, she started on alectinib with great response, and no liver function alteration recurred.

**Conclusions:**

This case suggests that alectinib, even belonging to the same drug class, could be used as an alternative agent when crizotinib is the etiology of liver damage, but more robust evidence has awaited.

## Background

Non-small-cell lung cancer (NSCLC) accounts for 80% of lung malignancies, the leading cause of cancer deaths worldwide. Unfortunately, the majority is already unresectable or metastatic upon its diagnosis [1] and will require systemic therapy. Adenocarcinoma is the most common NSCLC histologic subtype, and nowadays, its treatment relies on the molecular signature, tailored by specific driver mutations [2].

The anaplastic lymphoma kinase (ALK) fusion gene, found in 3–5% of NSCLC, is a driver mutation for which target therapies are available, such as crizotinib, first-in-class, multitargeted tyrosine kinase inhibitor (TKI) [3]. In patients with locally advanced or metastatic ALK-positive NSCLC, this oral drug showed improved survival compared to conventional chemotherapy [4–6].

Besides its clinical benefit, concerns about crizotinib hepatotoxicity have arisen. In the phase 3 trial PROFILE 1014, which granted the drug approval in Brazil and many other countries for ALK-positive NSCLC patients, 14% of patients in the crizotinib arm developed grade 3 transaminases elevation [5].

Since then, case reports have been published describing crizotinib potential liver injury and its management. However, it is not clear whether this is a drug class side effect or if the use of other selective ALKs inhibitors is safe after this severe toxicity.

Herein, we report a real-world case of acute hepatitis induced by crizotinib in an ALK-rearranged positive NSCLC patient, in whom the treatment shift for a second-generation ALK-inhibitor after the event recovery. A complete metabolic response was achieved, and no serious adverse events occurred.

## Case presentation

A 32-years-old female, non-smoker, had 3-months onset symptoms of dyspnea and cough. There were no comorbidities or medicine use. Her computer tomography (CT) scans showed multiple bilateral nodules associated with lymphangitis signs, enlarged bilateral mediastinal lymph nodes, and thoracic vertebral bone lesion. She underwent a right inferior lobe segmentectomy, whose path report showed a lung carcinoma. No brain metastasis was identified by MRI, and the PET-CT has not performed at initial staging.

As the symptoms were getting worse, the treatment based on carboplatin plus paclitaxel initiated, while complimentary histopathologic and molecular investigations have performed. After two cycles, the immunohistochemistry confirmed a pulmonary adenocarcinoma, and the ALK rearrangement (2p23q in more than 15% of the specimen) was detected by fluorescence in situ hybridization test. Moreover, PDL-1 was 40%, EGFR, ROS-1, MET, RET, ERBB2, and BRAF were negative.

Since the ALK-positive stage IV pulmonary adenocarcinoma diagnosis, the treatment has promptly adjusted for crizotinib—the only TKI approved in Brazil for this scenario at that moment—250 mg twice daily, on November 11th, 2018. After almost 60 days of therapy, despite respiratory symptoms improvement, the patient presented some non-specific complaints such as nausea and fatigue. Physical examination with no relevant finding, including jaundice. By December 21st, her laboratory review showed a severe liver dysfunction, shown in graph 1. Due to acute hepatitis, crizotinib therapy has halted. Viral involvement and other etiologies were investigated and excluded. Thenceforth, a gradual liver function recovery has occurred. On February 18th, 2019, blood tests did not show any significant alteration.

Meanwhile, during the period without specific oncology treatment, dyspnea and cough recurred, and the patient developed a headache onset. Central nervous system (CNS)—multiple small lesions along brain parenchyma—and lung progression were detected on February 4th.

**Fig. 1:**
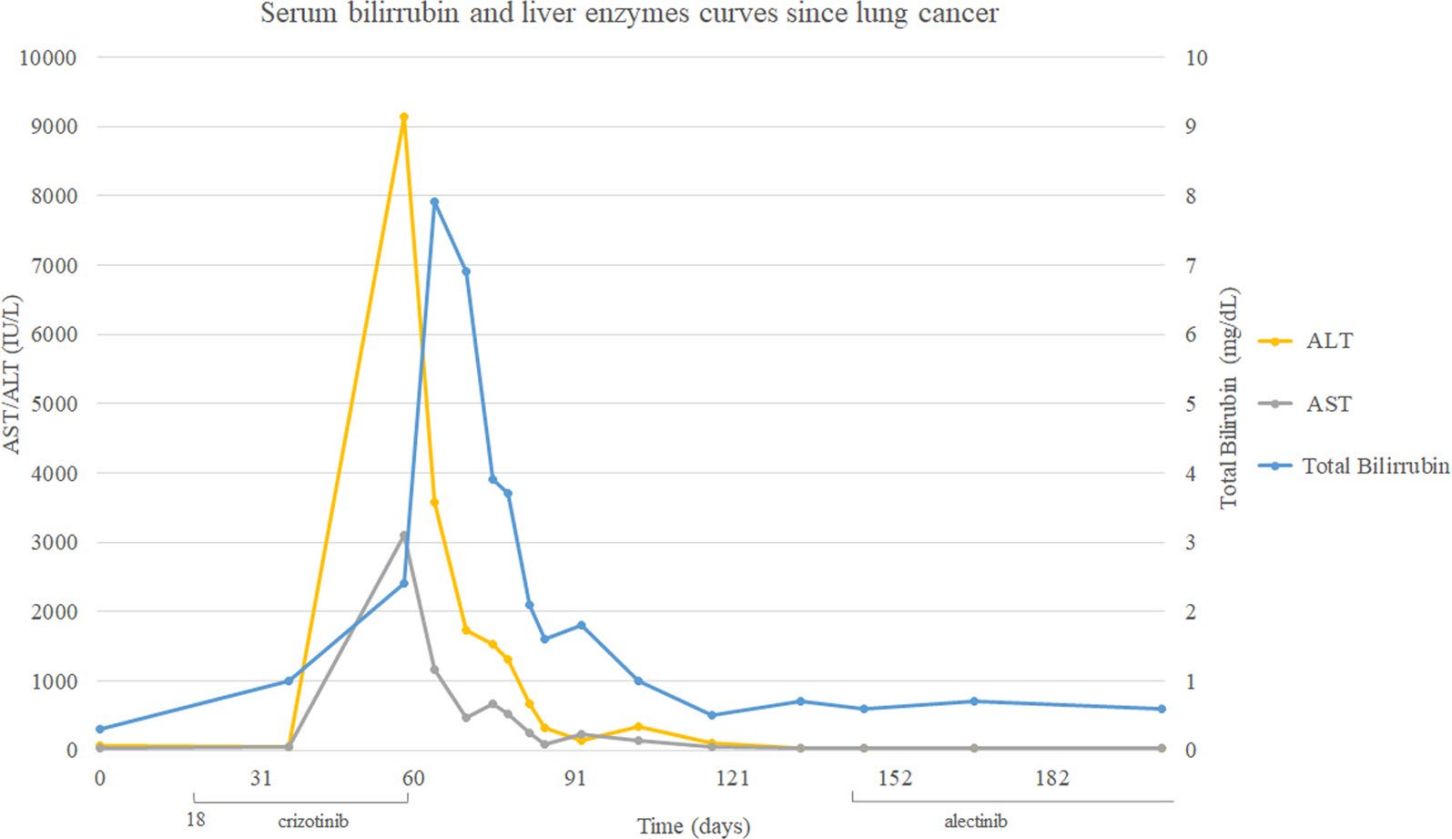
Serum bilirrubin and liver enzymes curves since lung cancer diagnosis

Since then, she started on alectinib 600 mg orally twice daily. A few weeks later, she completely recovered from her respiratory symptoms, and no liver function alteration recurred (see Fig. 1).

The patient is still under full dose alectinib therapy with excellent tolerability and no adverse effects. PET-CT performed on May 8th did not show any metabolic activity as well as the brain MRI did not show any evidence of CNS involvement.

## Discussion and conclusions

This report describes a successful case of treatment with alectinib after crizotinib-induced hepatitis. This serious adverse event may be ascribed to crizotinib due to the temporal relationship between drug beginning and the transaminases elevation and due to its resolution after the medication interruption.

So far, the mechanism of crizotinib liver toxicity is not clear, and specific risk factors or clinicopathologic predictors for crizotinib-induced liver injury have not yet been identified. The reported general risk factors of drug-induced hepatotoxicity include older age, female gender, HIV infection, HBV or HCV infection, pregnancy, excessive alcohol intake, smoking, and genetic variability [7, 8]. According to its prescribing information, the hepatotoxicity generally occurs within the first 2 months of the treatment, which was compatible with the case reported. Considerations about the pharmacodynamic properties are important regarding drug side effects. The liver metabolizes crizotinib, and CYP3A plays a major role. Therefore, we should avoid concomitant use of CYP3A inducers and inhibitors, which may alter crizotinib plasma concentrations [9]. However, there was no concomitant drug used by our patient.

Alectinib, a second-generation TKI targeting ALK, is also associated with elevations of AST and ALT, as showed by clinical trials. Among the 405 patients enrolled in intervention arms in NP28761, NP28673, and ALEX studies, AST and ALT elevations greater than five times the upper limit of normal (ULN) occurred in 4.6% and 5.3% respectively, bilirubin levels more than three times the ULN occurred in 3.7%. In the majority of patients, these events occurred in the first 3 months of treatment. Ten patients discontinued alectinib due to Grades 3–4 AST/ALT (n = 6) and bilirubin (n = 4) elevations. Thus, monitoring liver function tests, including ALT, AST, and total bilirubin every two weeks during the first 3 months of treatment, then once a month, or whenever clinically indicated, is strongly advisable [10].

This case report harbors some limitations. We did not perform a liver biopsy, and the association between the crizotinib with the liver damage was established only based on clinic and temporal criteria, which reflects real-world practice. Moreover, there is no specific recommendation regarding the use of alectinib after recovering from crizotinib-induced hepatitis. However, the patient had disease progression after crizotinib interruption, and the alectinib was the best option in the second-line setting at that time, based on phase II studies. At that time, other TKI targeted to ALK-rearrangement was not available in Brazil [11].

It is important to highlight the contribution of the present report: although the described occurrence of liver toxicity with both TKIs, hepatitis induced by one drug does not exclude the possibility of treatment with another specific ALK-TKIs.

While evidence from clinical trials is scarce, experiences like that, in a real-world scenario, may add to clinical decision. Once this class of drugs changed the natural history of the disease, its definitive discontinuation could impact the patient´s overall survival.

In conclusion, this case suggests that alectinib could be an alternative agent when crizotinib is the etiology of hepatitis. Therefore, patients might still derive benefit from target therapy.

## Data Availability

The data that support this case report are available from the corresponding author on reasonable request, since respecting the Ethics Committee to protect patient confidentiality.

## Abbreviations

NSCLC: Non-small cell lung cancer
ALK: Anaplastic lymphoma kinase
TKI: Tyrosine kinase inhibitor
CT: Computer tomography
CNS: Central nervous system
ULN: Upper limit of normal
